# The Crosstalks Between Jasmonic Acid and Other Plant Hormone Signaling Highlight the Involvement of Jasmonic Acid as a Core Component in Plant Response to Biotic and Abiotic Stresses

**DOI:** 10.3389/fpls.2019.01349

**Published:** 2019-10-18

**Authors:** Jing Yang, Guihua Duan, Chunqin Li, Lin Liu, Guangyu Han, Yaling Zhang, Changmi Wang

**Affiliations:** ^1^State Key Laboratory for Conservation and Utilization of Bio-Resources in Yunnan, Yunnan Agricultural University, Kunming, China; ^2^Key Laboratory of Agro-Biodiversity and Pest Management of the Ministry of Education, Yunnan Agricultural University, Kunming, China

**Keywords:** jasmonic acid, plant hormone, environmental stress, defense response, crosstalk

## Abstract

Plant hormones play central roles in plant growth, developmental processes, and plant response to biotic and abiotic stresses. On the one hand, plant hormones may allocate limited resources to the most serious stresses; on the other hand, the crosstalks among multiple plant hormone signaling regulate the balance between plant growth and defense. Many studies have reported the mechanism of crosstalks between jasmonic acid (JA) and other plant hormones in plant growth and stress responses. Based on these studies, this paper mainly reviews the crosstalks between JA and other plant hormone signaling in regulating the balance between plant growth and defense response. The suppressor proteins JASMONATE ZIM DOMAIN PROTEIN (JAZ) and MYC2 as the key components in the crosstalks are also highlighted in the review. We conclude that JA interacts with other hormone signaling pathways [such as auxin, ethylene (ET), abscisic acid (ABA), salicylic acid (SA), brassinosteroids (BRs), and gibberellin (GA)] to regulate plant growth, abiotic stress tolerance, and defense resistance against hemibiotrophic pathogens such as *Magnaporthe oryzae* and *Pseudomonas syringae*. Notably, JA may act as a core signal in the phytohormone signaling network.

## Introduction

Under multiple environmental stresses, plant hormones allocate limited resources to respond to the most serious stress (Tian et al., 2003; Matyssek et al., 2005) and develop various signaling pathways (Pieterse et al., 2012; Sharma et al., 2013) to regulate the balance between plant growth and defense response (Tian et al., 2003; Matyssek et al., 2005). Understanding the similarities and differences of plant hormone signaling may be important in agricultural production.

Plant hormones are small endogenous signaling molecules, including gibberellin (GA), auxin (indole-3-acetic acid, IAA), cytokinin (CK), brassinosteroids (BRs), abscisic acid (ABA), ethylene (ET), jasmonic acid (JA), salicylic acid (SA), and strigolactone (SL). In recent decades, JA biosynthesis has been widely investigated in monocotyledons and dicotyledons, especially in *Arabidopsis*. In *Arabidopsis*, at least two pathways are responsible for JA biosynthesis, namely, the α-linolenic acid (18:3) initial octadecane pathway and hexadecatrienoic acid (16:3) initial hexadecane pathway (Engelberth et al., 2001; Wasternack, 2014; Kazan, 2015). In these pathways, the 18:3 and 16:3 unsaturated fatty acids are converted to 12-oxo-phytodienoic acid (12-OPDA) and deoxymethylated vegetable dienic acid (dn-OPDA) in the chloroplast, respectively. Then JA is formed from 12-OPDA and dn-OPDA through multiple β-oxidation in the peroxisome. Finally, different structures of JAs such as methyl jasmonate (MeJA). JA–isoleucine (JA–Ile) and 12-hydroxyjasmonic acid (12-OH-JA) are formed from JA in the cytoplasm. Among these JAs, JA–Ile is the biological active form of JA in plants (Wasternack and Strnad, 2016).

JA is widely distributed in plants as a natural plant growth regulator (Engelberth et al., 2001; Ghasemi Pirbalouti et al., 2014; Wasternack, 2014; Kazan, 2015; Ahmad et al., 2016; Wasternack and Strnad, 2016). The importance of the crosstalks between JA and other plant hormones in regulating plant stress responses has attracted extensive attention (Kazan, 2015; Ahmad et al., 2016; Wasternack and Strnad, 2016; Wasternack and Song, 2016). In this paper, the role of the crosstalks between JA and other plant hormone signaling in regulating plant stress responses as well as in the balance of plant growth and defense response is reviewed.

## Core Components of JA Signaling

### SKP1/CULLIN/F-Box (SCF)^COI1^ Complex

CORONATINE INSENSITIVE 1 (COI1) is assembled into SCF E3 ubiquitin ligase complex SCF^COI1^ to be stabilized (Zhang et al., 2015). As an F-box protein and a component of SCF^COI1^, COI1 plays an important role in recognizing JA signaling (Xie et al., 1998). In the JA signaling pathway, the F-box protein is recognized and combined with targeted proteins, and then the targeted proteins are degraded by 26S proteasome.

## JASMONATE ZIM DOMAIN (JAZ) Protein: Inhibitor of JA Response

JAZ protein contains two conserved protein–protein interaction domains, ZIM and Jas (Thireault et al., 2015). The Jas domain of JAZ protein mediates the interaction between JAZ and COI1 or other transcription factors (TFs) (Gimenez-Ibanez et al., 2015), the ZIM (TIFY) domain of JAZ protein mediates JAZ dimerization and its interaction with NINJA, and NINJA recruits general transcriptional co-suppressor TPL through the conserved EAR domain. Moreover, it competes with MEDIATOR25 (MED25) to interact with MYCs (Zhang F et al., 2015).

## MYC TFS

In contrast to the positive regulation of JAZ in plant growth, MYC TFs (MYC2, MYC3, and MYC4) negatively regulate gene expression in cell cycle, thereby inhibiting plant growth (Gasperini et al., 2015; Campos et al., 2016; Major et al., 2017). As the general switch of the JA signaling pathway, MYC2 is the most famous TF. Moreover, it participates in the crosstalks among JA, ABA, auxin, ET, GA, and other signaling pathways (Gangappa et al., 2013).

### Roles of SCF^COI1^ Complex, MYC2, and JAZ in JA Signaling Pathway

**Figure 1:**
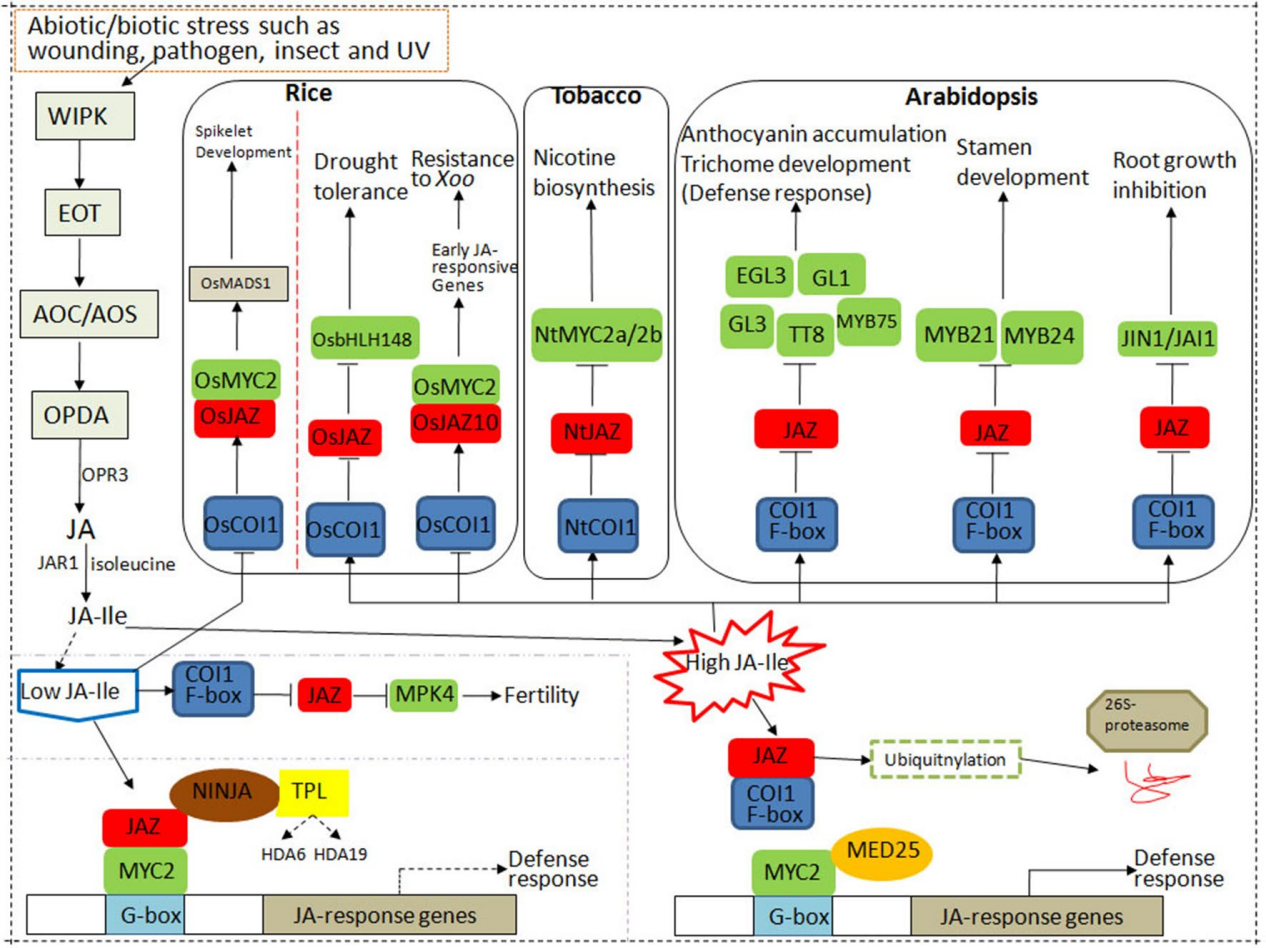
The core components of the jasmonic acid (JA) signaling pathway in rice, tobacco, and *Arabidopsis*. CORONATINE INSENSITIVE 1 (COI1) protein, JASMONATE ZIM DOMAIN PROTEIN (JAZ), and MYC constitute the core signal transduction mechanism of JA signaling. Under control conditions, the endogenous level of JA–isoleucine (Ile) is very low plants. JAZ repressors bind to MYC2 to inhibit its transcriptional activation on downstream genes. Under stress conditions, the endogenous level of JA–Ile is largely activated, which is perceived by JA receptor COI1. Then SKP1/CULLIN/F-box (SCF)^COI1^ binds to JAZs for ubiquitination and degradation through the 26S proteasome pathway, resulting in the release of the downstream transcription factors (TFs) such as MYCs and the activation of JA responses.

COI1 protein, JAZ, and MYC constitute the core signal transduction mechanism of JA signaling and have been proven to be the intersection of other signal transduction pathways under various stresses (**Figure 1**). JAZ and various TFs form specific JAZ/TFs that specifically regulate multiple downstream responses (Chini et al., 2016). The JAZ-MYC module increases the concentration of defense compounds to trigger defense response or inhibit plant growth against pathogen infection (Havko et al., 2016). In addition to the JAZ-MYC module, COI1-JAZ2-MYC2, 3,4-ANAC19,55,72 (Gimenez-Ibanez et al., 2017), and other specific JAZ-TF modules have been identified (Jin and Zhu, 2017; Mao et al., 2017). The interaction between MYCs and JAZs may involve other plant hormone signaling pathways such as ET-mediated cell division through ET RESPONSE FACTOR (ERF) TFs (Zhang F et al., 2015; Gimenez-Ibanez et al., 2017; Dubois et al., 2018).

When JA–Ile content is high in rice, OsCOI1 interacts with OsJAZ to form a complex, JAZ protein is degraded, and the transcription of OsbHLH148 in the JA signal module is rapidly activated to induce drought tolerance. High levels of JA–Ile induce OsMYC2 to selectively bind to the G-box-like motif in the promoter region of OsJAZ10 to activate transcription activity, induce the expression of early JA-responsive genes, and thereby resist bacterial pathogens (Uji et al., 2016).

Generally, the endogenous level of biologically active JA (JA–Ile) is kept very low plants but can be rapidly activated in response to various stresses such as insect feeding or wounding. Then JA signaling is perceived by JA receptor COI1, an important component of SCF^COI1^, which binds to JAZs for ubiquitination and degradation through the 26S proteasome pathway. The competitive binding and degradation of JAZ repressors can further release the downstream TFs such as MYCs, resulting in the activation of JA responses by MYCs (Gimenez-Ibanez et al., 2017; Dubois et al., 2018).

To sum up, the three main core components of JA signaling play an important role in plant growth, development, and response to biotic or abiotic stresses. When exogenous JA or extreme stress is applied, the concentration and application time may affect the transcriptional activities of different components in the JA signal module. Therefore, according to the JA metabolic pathway under stress conditions, corresponding stress-tolerance breeding may be developed to improve crop resistance in agriculture.

## Crosstalks Between JA and Other Plant Hormone Signaling Pathways

**Figure 2:**
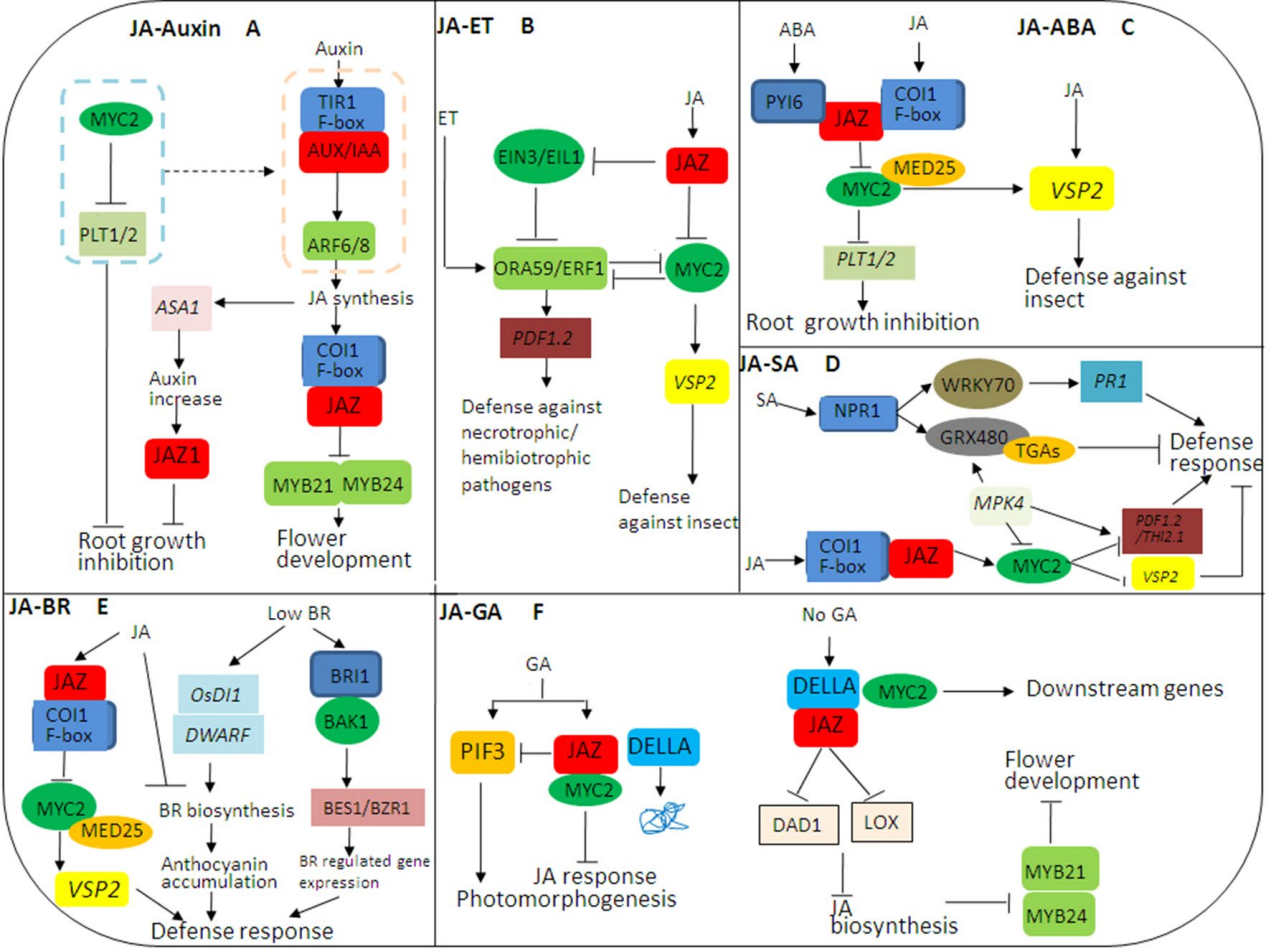
JASMONATE ZIM DOMAIN PROTEIN (JAZ)-mediated crosstalks among jasmonic acid (JA) hormone signaling pathways in plant growth and stress responses. **(A)** The complex crosstalk between JA and auxin signaling pathways. JA and auxin signaling pathways coordinately regulate flower development through modulation of JA, while JA and auxin antagonize root growth through JAZs-MYC2. **(B)** The complex crosstalk between JA and ethylene (ET) signaling pathways. JA and ET coordinately regulate plant stress responses through JAZs-MYC2 and EIN3/EIL1, especially in resisting necrotrophic or hemibiotrophic pathogens. **(C)** The complex crosstalk between JA and abscisic acid (ABA) signaling pathways. The crosstalk between PYRABACTIN RESISTANCE1-Like protein (PYL) and JAZ–MYC2 coordinates the balance between plant growth and defense resistance. **(D)** The complex crosstalk between JA and SA signaling pathways. SA initiates early defense-related gene expression in pathogen-infected plants, while JA induces late defense-related gene expression in pathogen-infected plants, mainly in the necrotrophic stage of necrotrophic or hemibiotrophic pathogens. **(E)** The complex crosstalk between JA and brassinosteroids (BR) signaling pathways. The crosstalk between JA and BR biosynthesis may be involved in the balance between plant growth and defense resistance. **(F)** The complex crosstalk between JA and GA signaling pathways. The JAZ-MYC2-DELLA-PIF signaling module being involved in the crosstalk between JA and GA signaling can be elucidated. In addition, many transcription factors (TFs) such as MYC3, MYC4, MYB21, and MYB24 can also interact with DELLAs, so there may be synergistic effect between JA and GA signaling.

The crosstalk between plant hormones is the core of plant stress response (He et al., 2017). JA does not work independently but acts in a complex signaling network combined with other plant hormone signaling pathways (Kazan, 2015; Ahmad et al., 2016; Wasternack and Strnad, 2016; Hu et al., 2017). As a core component of JA signaling, the role of JAZs-MYC2 in the crosstalks of plant hormone signaling pathways is highlighted in this review, especially in the crosstalks of JA–auxin, JA–ET, JA–ABA, JA–SA, JA–BR, and JA–GA signaling pathways (Pérez and Goossens, 2013; Gimenez-Ibanez et al., 2015; Chini et al., 2016; Goossens et al., 2016) (**Figure 2**).

### JA–Auxin Crosstalk

JA and auxin signaling pathways coordinately regulate plant growth and development. COI1, MYC2, and JAZ, as the main core components, participate in the crosstalk of JA and auxin signaling pathways (**Figure 2A**). When plants are induced by exogenous auxin, the auxin–TIR–AUX/IAA–ARF signaling is activated, and JA synthesis is induced. On the one hand, the endogenous JA induces the expression of auxin synthase gene (*ASA1*) and auxin content, so JA regulates the biosynthesis of auxin and further regulates the expression of JAZ1 and root growth. On the other hand, JA induces the formation of a complex of COI1 and JAZ and leads to the degradation of JAZ, thereby activating the transcriptional activities of MYB21/MYB24 and inducing flower development. Notably, MYC2 inhibits the expressions of *PLETHORAs* (*PTL1* and *PTL2*) and counteracts the auxin–TIR–AUX/IAA–ARF signaling, so as to regulate root growth (Chen et al., 2011). In addition, ARF6/ARF8 in the auxin signaling pathway regulates petal and stamen growth through modulation of endogenous JA level, and MYB21/MYB24 as downstream of JAZ in JA signaling pathways also coordinately regulate petal and stamen growth (Reeves et al., 2012). Thus, JA and auxin signaling pathways coordinately regulate flower development through modulation of JA, while JA and auxin antagonize root growth through JAZs-MYC2.

### JA–ET Crosstalk

JA and ET antagonize or coordinately regulate plant stress response (Zhu, 2014; Zhu and Lee, 2015) (**Figure 2B**). ET INSENSITIVE3 (EIN3) and its homologue EIN3-like 1 (EIL1) in the ET signaling pathway as well as JAZs-MYC2 in the JA signaling pathway are involved in the crosstalk between JA and ET signaling pathways (Zhang et al., 2014). On the one hand, exogenous JA triggers the degradation of JAZ, and the release of MYC2 regulates the expression of ORA59/ERF1 and wound responsive gene *VSP2*, so as to resist herbivorous insects. On the other hand, JAZ inhibits the transcriptional activity of EIL2/EIN3 in the ET signaling pathway and activates downstream ORA59/ERF1 that targets the promoter of *PLANT DEFENSIN 1.2* (*PDF1.2*) and induces its expression, thereby resisting the infection of necrotrophic pathogens and hemibiotrophic pathogens (Zhu et al., 2011). Generally, the JA signaling pathway synergistically crosstalks with the ET signaling pathway against necrotrophic pathogen attacks and activates the expression of defense proteins such as PDF1.2 through ERF1 and ORA59. Thus, JA and ET coordinately regulate plant stress responses through JAZs-MYC2 and EIN3/EIL1, especially in resisting necrotrophic or hemibiotrophic pathogens (Pieterse et al., 2012).

### JA–ABA Crosstalk

ABA and JA signaling pathways coordinately regulate plant response to herbivorous insect feeding while antagonizing plant growth and development. JAZs-MYC2 participates in the crosstalk between JA and ABA signaling pathways, affecting plant growth and defense (Chen et al., 2011). ABA receptor PYRABACTIN RESISTANCE1-Like proteins (PYLs) regulate metabolic reprogramming in *Arabidopsis thaliana* and tobacco through the JA signaling pathway. Therefore, the crosstalk between ABA and JA signaling pathways can monitor elicitor-induced reprogramming of plant metabolism and growth (Per et al., 2018). ABA receptor PYL forms a complex with JAZ, which activates the transcriptional activated activity of MYC2. On the one hand, MYC2 activates the expression of JA responsive gene *VSP2* under the mediation of MED25 to resist herbivorous insect feeding. On the other hand, MYC2 inhibits the expressions of *PTL1* and *PTL2* as well as root growth. Additionally, ABA initiates the degradation of JAZ12, which plays a specific role in the crosstalk between JA and ABA signaling pathways (Pauwels et al., 2015). Thus, the crosstalk between JA and ABA signaling, especially between PYL and JAZ-MYC2, coordinates the balance between plant growth and defense resistance (**Figure 2C**).

### JA–SA Crosstalk

Generally, JA is widely involved in regulating disease resistance against necrotrophic pathogens, while SA mediates broad-spectrum resistance against biotrophic and hemibiotrophic pathogens (Fu et al., 2012). It has been shown that JA signaling can inhibit SA accumulation through modulation of multiple NAC TFs, such as ANAC019/055/072. Briefly, MYC2 directly binds to the promoters of these NACs and then activates their transcription. Then the activation of these NAC TFs further inhibits the expression of ISOCHORISMATE SYNTHASE 1 (ICS1) as an SA biosynthesis gene while triggering the expression of BENZOIC ACID/SA CARBOXYL METHYLTRANSFERASE 1 (BSMT1) as an SA methylation gene (Zheng et al., 2012). In addition, the crosstalk between JA and SA signaling pathways involves many components, including mitogen-activated protein kinase (MAPK) (Rodriguez et al., 2010); redox regulators glutathione (GRX) and thioredoxin (TRX) (Spoel and Loake, 2011), MYC2, TGAs, and PDF 1.2 (Gatz, 2013); and WRKY70 (Shim et al., 2013). In the presence of exogenous SA, NONEXPRESSOR OF PR GENES1 (NPR1) is activated to induce the transcriptional-activated activity of WRKY70, which promotes the expression of *PR1* by binding to the promoter region of *PR1* and inducing defense response. In the meanwhile, NPR1 polymers are monomerized by TRX through SA-induced redox state changes, and then monomers such as GRX480 are transported to the nucleus and specifically bind to TGAs, which also directly regulate the expression of *PR1* (Leon-Reyes et al., 2009; Spoel et al., 2009; Fu et al., 2012; Zander et al., 2012; Gatz, 2013) (**Figure 2D**). Thus, the transformation between NPR1 polymer and monomer has a dual role in inhibiting and activating defense-related gene expression (Wasternack and Hause, 2013). Interestingly, the induction of GRXs can block TGA-mediated JA response gene expression, such as ORA59, further confirming SA–JA antagonism (Zander et al., 2012). MPK4 positively regulates GRX480 in the SA signaling pathway and negatively regulates MYC2 in the JA signaling pathway, which is necessary for JA responsive genes (*PDF1.2* and *THI2.1*) (Wasternack and Hause, 2013). Therefore, MYC2 and its upstream MPK4 are involved in the crosstalk between JA and SA signaling pathways, which coordinately regulate plant disease resistance against necrotrophic or hemibiotrophic pathogens. SA initiates early defense-related gene expression in infected plants, while JA induces late defense-related gene expression in infected plants, mainly in the necrotrophic stage of necrotrophic or hemibiotrophic pathogens.

### JA–BR Crosstalk

JA inhibits plant growth, while BR induces above-ground plant growth. The crosstalk between JA and BR signaling pathways is involved in the balance between plant growth and defense resistance. On the one hand, low concentration of BR induces the expression of *OsDI1* and *OsDWARF* at the early and late stages of BR biosynthesis, respectively, and anthocyanin accumulation and activates defense response. On the other hand, high concentration of BR activates BR signaling cascades including BR receptor BRI1, BR-related kinase BAK1, and BR-related TFs to induce the expressions of downstream genes such as *BES1* and *BZR1*, thereby regulating plant responses to abiotic stresses (**Figure 2E**). JA induces JAZ to bind COI1, and MYC2 activates the expression of *VSP2* under the mediation of MED2, thereby resisting herbivorous insect feeding (**Figure 2E**). Notably, high concentration of BR inhibits endogenous biosynthesis of JA and BR, and JA also inhibits BR biosynthesis (Choudhary et al., 2012). Thus, the crosstalk between JA and BR biosynthesis may be involved in the balance between plant growth and defense resistance.

### JA–GA Crosstalk

JA and GA signaling pathways coordinately and antagonistically regulate plant growth and defense response; however, plant defense response is exerted at the cost of inhibiting growth (Yang et al., 2012; Wasternack and Hause, 2013). The C-terminus of JAZs is necessary for the interaction between JAZs and MYC2 and between JAZs and DELLAs, so DELLAs can completely interact with JAZs (Hou et al., 2010). In the absence of GA, stable DELLA interacts with JAZ to release MYC2, resulting in the activation of MYC2 downstream genes. At the same time, DELLA interacts with JAZ to inhibit the expression of JA biosynthetic genes (*DAD1* and *LOX*) and further inhibits JA biosynthesis as well as the activities of MYB21 and MYB24 (**Figure 2F**), thereby regulating stamen development (Song et al., 2011). GA induces the degradation of DELLA and the binding of JAZ to MYC2, so as to inhibit JA signaling. In addition, GA induces PIF3/PIF4 to regulate photomorphogenesis (**Figure 2F**) (Hou et al., 2013). Notably, JA delays GA-mediated degradation of DELLA, the *della* mutant is less sensitive to JA-inhibited plant growth inhibition, and AtJAZ9 inhibits the interaction between DELLA and PIF3 (Yang et al., 2012). Therefore, the molecular cascade involving the JAZ-MYC2-DELLA-PIF signaling module in the crosstalk between JA and GA signaling pathways can be elucidated. Moreover, many TFs such as MYC3, MYC4, MYB21, and MYB24 can also interact with DELLAs, so there may be a synergistic effect between JA and GA signaling pathways (Lyons et al., 2013).

## JA Regulates Plant Response to Biotic/Abiotic Stresses

The role of JA in plant response to abiotic stresses has been extensively reported (Khan et al., 2012; Kazan, 2015; Ahmad et al., 2016; Sharma and Laxmi, 2016; Wasternack and Strnad, 2016; Per et al., 2018), such as heavy metals (Maksymiec et al., 2005), drought (Brossa et al., 2011), salt (Qiu et al., 2014; Zhao et al., 2014), heat (Clarke et al., 2009), and UV radiation (Liu et al., 2012). The role of JA in plant response to biotic stress is mainly to induce plant disease response against pathogen or insect (Campos et al., 2014). Under pathogen infection conditions, JA and JA–Ile can be rapidly induced (Chauvin et al., 2013; Fragoso et al., 2014), which induces almost all major secondary metabolites and protein expression involved in defense response, including alkaloids, terpenoids, phenylpropane, amino acid derivatives, anti-nutritional proteins, and some pathogen-related proteins (De et al., 2012; De et al., 2013). In this paper, the effects of JA on plant response to abiotic/biotic stresses including cold stress, drought stress, and fungal and bacterial diseases are discussed.

### JA and Cold Stress

**Figure 3:**
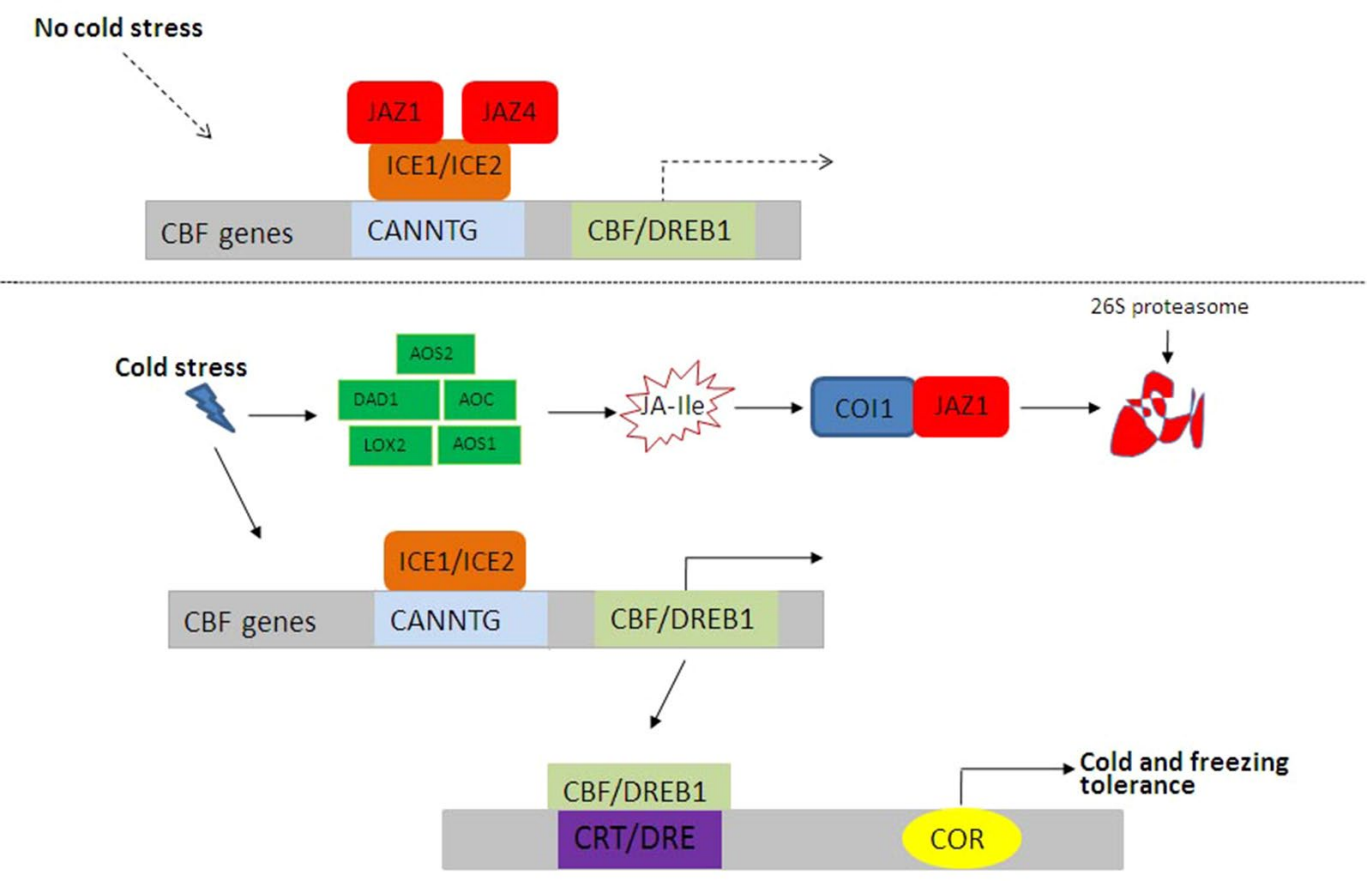
JA-mediated cold and freezing stress responses in plants. Under control conditions, JAZ1 and JAZ4 interact with ICE1 and ICE2 to inhibit the ICE–CBF signaling pathway. Under low-temperature conditions, bioactive JA–Ile and ICE–CBF pathways are activated, and the expressions of cold-regulated genes are induced to improve plant cold tolerance. CBF, C repeat binding factor; ICE, INDUCER OF CBF EXPRESSION; Ile, isoleucine; JA, jasmonic acid; JAZ, JASMONATE ZIM DOMAIN PROTEIN.

In order to adapt to extremely low temperatures, plants have evolved complex mechanisms by regulating physiological and biochemical processes, especially the modulation of stress-related gene expression. The INDUCER OF CBF EXPRESSION (ICE)-CBF transcriptional cascade signaling pathway plays a central role in plant cold stress response (Hu et al., 2017). ICE1 and ICE2, two basic helix–loop–helix (bHLH) TFs in *A. thaliana*, upregulate the expressions of *CBFs* through directly binding to CANNTG in the promoter region of *CBFs*. Rice grows at normal temperature. Under control conditions, JAZ1 and JAZ4 interact with ICE1 and ICE2 to inhibit the ICE-CBF signaling pathway (**Figure 3**). Under low-temperature conditions, the expressions of JA synthesis-related genes including *allene oxide synthase1* (*AOS1*), *DAD1*, *allene oxide cyclase* (*AOC*), *LOX2*, and *AOS1* are induced (Zhu, 2016; Hu et al., 2017), and bioactive JA–Ile is synthesized, thereby activating JA receptor COI1 to bind to JAZ1, resulting in the degradation of JAZ1 through 26S proteome after ubiquitination (**Figure 3**). Then, the ICE-CBF transcriptional regulation cascade signaling pathway is activated, and the expressions of cold-regulated genes are induced to improve plant cold tolerance. Moreover, JA–ABA crosstalk (Wang et al., 2016) and JA–GA crosstalk in plant response to cold stress have been extensively studied (Achard et al., 2008).

### JA and Drought Stress

**Figure 4:**
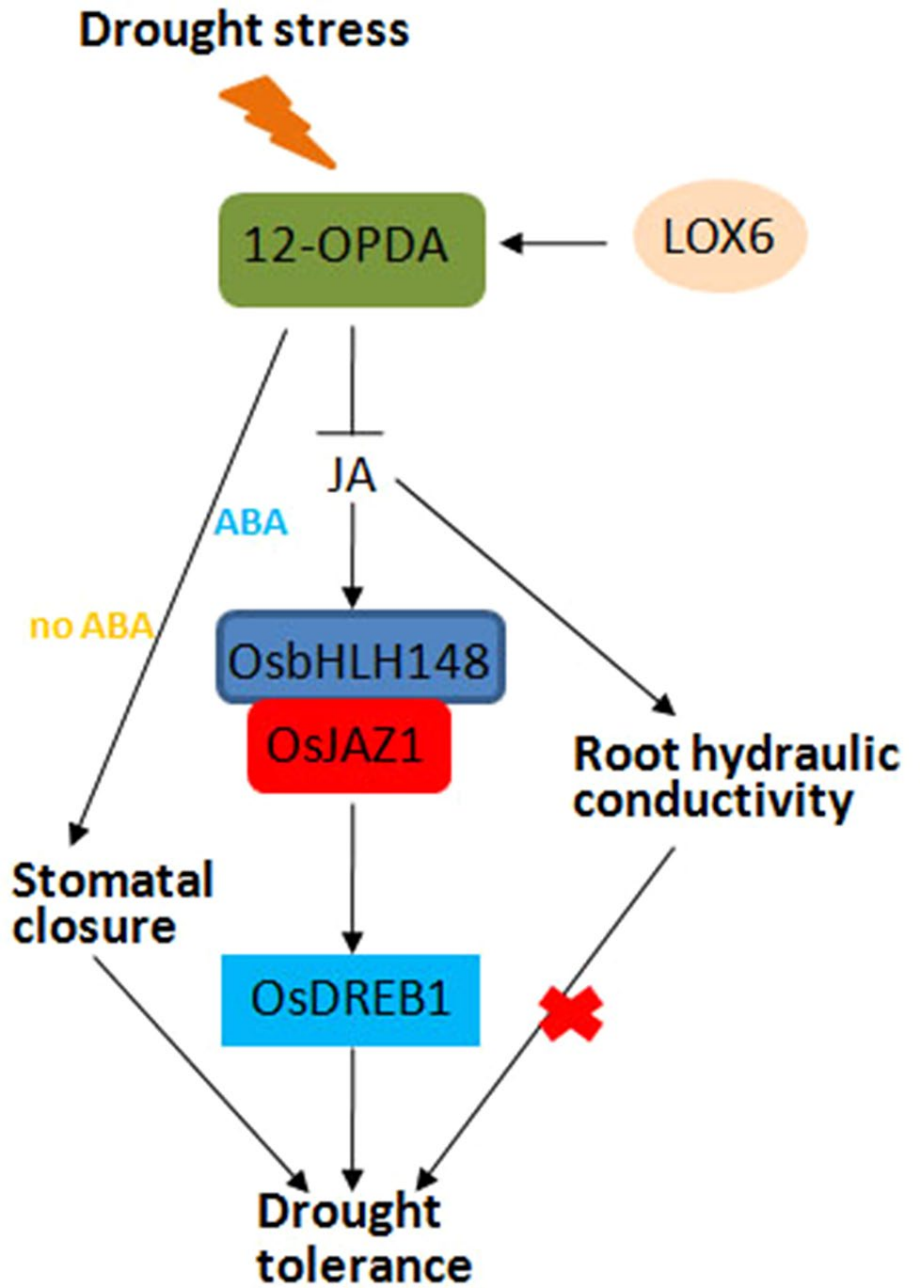
Jasmonic acid (JA)-mediated drought stress response in plants. In response to drought stress, the JA signaling pathway is activated; OsbHLH148 interacts with OsJAZ1 to activate the expression of *OsDREB1*, together with JA-mediated root hydraulic conductivity and stomatal closure, thereby improving drought tolerance in rice.

Drought stress response is a complex process in plants. Stomatal closure can reduce water loss and is a potential drought resistance mechanism of plants (Acharya and Assmann, 2009). JA and JA precursor 12-OPDA can promote stomatal closure in *A. thaliana*, and the increase of OPDA content is related to the decrease of stomatal aperture and improved drought resistance (Savchenko et al., 2014). 13-Lipoxygenase LOX6 is essential for the synthesis of 12-OPDA and plays an important role in plant drought tolerance. When drought stress is applied, LOX6 is synthesized to 12-OPDA, which promotes stomatal closure and improves drought tolerance in the presence or absence of ABA (**Figure 4**). In response to drought stress, the JA signaling pathway is activated, and OsbHLH148 interacts with OsJAZ1 to activate the expression of *OsDREB1*, thereby improving drought tolerance in rice (**Figure 4**) (Sarwat and Tuteja, 2017). Moreover, some antioxidant enzymes, including superoxide dismutase (SOD), peroxidase (POD), catalase (CAT), proline, and relative water content (RWC), are increased to enhance the ability of plants to cope with drought stress (Sarwat and Tuteja, 2017).

### JA and Fungal Diseases

**Figure 5:**
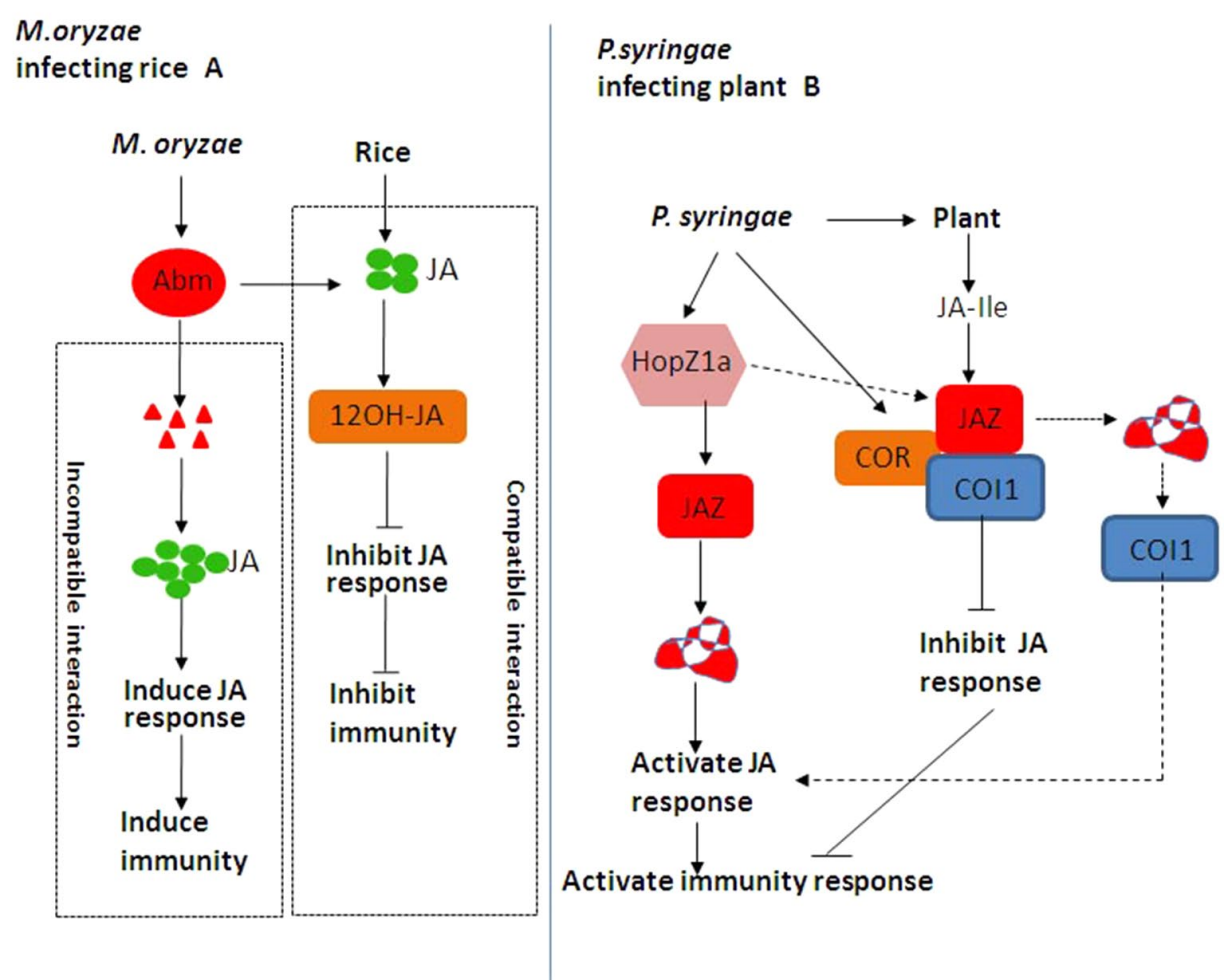
**(A)** Jasmonic acid (JA)-mediated disease resistance against *Magnaporthe oryzae* in rice. When rice blast fungus is compatible with rice, rice blast fungus secretes antibiotic biosynthetic monooxygenase (Abm) and inhibits JA activity and immune response. When rice blast fungus and rice are incompatible, Abm secreted by rice blast fungus is degraded, resulting in the accumulation of methyl jasmonate (MeJA) and the activation of JA downstream response as well as immune response. **(B)** JA-mediated disease resistance against *Pseudomonas syringae* in *Arabidopsis*. HopZ1a directly interacts with JASMONATE ZIM DOMAIN PROTEIN (JAZ) proteins and induces the acetylation of JAZ proteins, thereby activating the JA signaling pathway (Jiang et al., 2013). As one kind of functional JA analog, coronatine (COR) can induce CORONATINE INSENSITIVE 1 (COI1) to bind to JAZ proteins, thereby activating JA downstream response and plant immune response (Zhang F et al., 2015).

Hemibiotrophic and necrotrophic fungi have a wide host range, resulting to serious yield losses in many important crops (Mengiste, 2012; Pandey et al., 2016). JA plays an important role in inducing plant against necrotrophic and hemibiotrophic pathogen and herbivorous insect feeding (Hu et al., 2013; Okada et al., 2015; Pandey et al., 2016). Some hemibiotrophic fungi can metabolize JA produced by host plants. The antibiotic biosynthetic monooxygenase (Abm) formed by *Magnaporthe grisea* can convert JA into 12-OH-JA to weaken JA signaling and promote the colonization of *Magnaporthe oryzae* (Patkar et al., 2015; Zhang et al., 2017). When rice blast fungus is compatible with rice, it secretes Abm and inhibits JA activity and immune response (**Figure 5A**). When rice blast fungus and rice are incompatible, Abm secreted by rice blast fungus is degraded, resulting in the accumulation of MeJA and the activation of JA downstream response as well as immune response (**Figure 5A**). So far, the JA–MYC2–PDF1.2 module is widely involved in plant–fungi interaction (Zhang et al., 2017). With the effect on promoting pathogenesis, the expression of LOB DOMAIN-CONTAINING PROTEIN 20 (LBD20) is closely related to *VSP2* and *THIONIN 2.1* (*Thi2.1*) as well as MYC2 (Thireault et al., 2015). MED19a is an important member of the mediator co-activator complex in JA signaling, and it can be degraded by an oomycete effector protein HaRxL44 (Caillaud et al., 2013). We have to note that the underlying mechanism of the JA signaling pathway in plant–fungi interaction remains elusive and needs to be further investigated.

### JA and Bacterial Diseases

Many pathogenic variants of hemibiotrophic bacteria *Pseudomonas syringae* can produce polyketide toxin coronatine (COR), AvrB, and HopZ1a. The most widely known example of JA-mediated plant–pathogen interaction is regulated by COR. COR is an active substance similar to JA–Ile in structure and function, with two moieties including coronamic acid and coronafacic acid (Bender et al., 1999). On the one hand, COR can promote bacterial infection through the modulation of pathogen-associated molecular pattern-triggered immunity (PTI)-activated stomatal closure and defense response (Sheard et al., 2010; Zhang L et al., 2015). On the other hand, COR can directly bind to the COI1–JAZ complex, and the activation of COR-mediated JA signaling pathway inhibits SA-mediated plant defense resistance against *P. syringae* infection (Zeng and He, 2010; Zhang L et al., 2015). In addition, COR has toxic functions by regulating secondary metabolites and inhibiting callose formation, which is independent of plant hormone antagonism (Millet et al., 2010; Geng et al., 2012; Yi et al., 2014). Therefore, JA–Ile mimics such as COR may be essential for the infection of some bacterial pathogens. COR can also enhance the interaction between COI1 and JAZ proteins (Zheng et al., 2012; Zhou et al., 2015; Zhang L et al., 2015).

AvrB regulates JA signaling through modulation of the COI1-dependent manner in *Arabidopsis* (He et al., 2004). In this case, the *Arabidopsis* protein RPM1-INTERACTING PROTEIN 4 (RIN4) appears to be involved (Cui et al., 2010; Zhou et al., 2015). AvrB interacts with RIN4 and triggers the plasma membrane-localized AHA1. Both AvrB and AHA1 promote the interaction between COI1 and JAZ, thereby regulating stomatal opening and plant defense response (Zhou et al., 2015). Unlike COR and AvrB, as an acetyl transferase, HopZ1a directly interacts with JAZ proteins and induces the acetylation of JAZ proteins, thereby activating the JA signaling pathway (Jiang et al., 2013) (**Figure 5B**).

### Role of Crosstalks Between JA and Other Plant Hormones in Plant Growth and Defense Balance

The crosstalks between plant hormone signaling pathways promote the balance between plant growth and defense (Huot et al., 2014). In order to survive and reproduce, plants should not only maintain growth but also resist pathogen infection. Therefore, the balance between plant growth and defense resistance has important ecological, agricultural, and economic values. The JAZ-MYC module in the JA signaling pathway plays a central role in the balance by integrating TF complexes and plant metabolic pathways (Guo et al., 2018).

When plants are infected by pathogens, PTI as the first defense system of plants, is activated rapidly, followed by SA, JA, and other plant hormone signaling pathways. In the meanwhile, auxin, BR, and GA signaling pathways related to plant growth are inhibited (Boller and He, 2009; Dou and Zhou, 2012). The changes in the amount and composition of stress-related hormones promote plant defense response (Denancé et al., 2013; Xie et al., 2016). When plants are subjected to biotic stress, the transient PTI response and the subsequent SA, JA, GA, BR, and other plant hormone signaling pathways have a certain persistence (Pieterse et al., 2012). SA signaling is mainly involved in disease resistance against biotrophic pathogens, while JA signaling is mainly involved in disease resistance against necrotrophic pathogens or the necrotrophic stage of hemibiotrophic pathogens (Spoel et al., 2007). The crosstalk between GA and JA signaling pathways plays a major role in balancing plant growth and defense against biotic and abiotic stresses (Hou et al., 2010; Wild et al., 2012; Yang et al., 2012; Heinrich et al., 2013; Hou et al., 2013; Daviere and Achard, 2016). GA regulates many aspects of plant growth, and JA plays a major role in stress response. JA and BR coordinately regulate plant environmental stresses, while JA and BR antagonize plant growth (Checker et al., 2018). BR negatively regulates PTI response, because the inhibition of PTI-induced gene expression may lead to the decrease of BR biosynthesis (Jiménez-Góngora Tamara et al., 2015). In summary, the balance between plant growth and defense disease depends on the crosstalks between PTI, JA, SA, BR, and GA signaling pathways.

## Prospect

In nature, plants are often subjected to various biotic and abiotic stresses. In response to these stresses, plants initiate a series of defense responses, PTI and effector-triggered immunity (ETI); among them are main defense responses (Katagiri and Tsuda, 2010). The plant hormone signaling network also plays an important role in the early regulation of plant defense response as well as plant–pathogen interaction. Hemibiotrophic fungi have a biotrophic stage, biotrophy-to-necrotrophy switch, and necrotrophic stage; the infected strategies are different at different stages, so it is necessary to further investigate whether plant hormones play the same or different roles at the whole stage of infection (Bhadauria et al., 2013; Chowdhury et al., 2017). Because plant hormones are natural and nontoxic in plants and the crosstalks among them regulate the balance between plant growth and defense resistance, it may have a broad application prospect to develop plant hormones as safe and environment-friendly elicitors by utilizing the crosstalks between plant hormone signaling pathways.

In recent decades, although the JA signaling pathway has been extensively investigated, the current understanding of its role in different environmental stresses is limited, due to the complex networks and crosstalk between multiple stresses and multiple signaling pathways. So far, the molecular mechanism of JA signaling in stress responses remains elusive. Compared with unidentified components, the identified components in plant hormone signaling pathways are limited. In addition, so many receptors and kinases exist in the cell membrane, and different environmental stresses may activate multiple enzymes, with a series of activation of secondary messengers such as Ca^2+^ and the reaction of kinase-TF-downstream genes. At present, there are still a lot of questions or gaps in understanding the crosstalks between JA and other hormones in plant stress responses, especially in the perception of multiple environmental signals. With the development of protein interaction omics, the complex protein interaction network may provide more clues to the understanding of complex stress signaling perception and protein complex-mediated plant hormone crosstalks.

The plant hormone signaling network is complex and changeable. Although “omics” methods have achieved global analyses of gene expression and protein spectrum changes to some extent, the data still fail to fully understand the dynamic spatial and temporal processes of plant hormone signaling networks in the balance between plant growth and defense resistance (Huot et al., 2014). Moreover, data in the lab may be largely different from those in the field, providing limited information for agriculture production. Therefore, it is necessary to comprehensively analyze plant hormone signaling networks during the whole developmental stages in the field; this will provide more values for crop breeding in the future. In addition, there are many questions that have remained unclear so far. For example, how do plant hormones regulate various plant responses to multiple environmental stress at the same time? How can plant hormones be effectively used to improve crop quality and stress tolerance in agricultural production? Therefore, we have to notice that the current understanding remains limited compared with unknown questions; further investigation will provide a novel insight into developing plant hormones for agricultural production by improving stress resistance and crop quality.

## Author Contributions

JY, GD, CL, LL, GH, YZ, and CW conceived the topic. GD, CL, and LL collected the data about jasmonate acid and crosstalking of JA and other plant hormones. GH, YZ, and CW collected the data about abiotic and biotic stresses. JY performed the network analysis and worked with other co-authors to complete the manuscript.

## Funding

The work was supported by grants from the national Key Research and Development Program of China (2016YFD100600) and NSFC (31860483 and 31400073) and Program for Innovative Research Team (in Science and Technology, IRTSTYN) in the University of Yunnan Province and State Key Laboratory for Conservation and Utilization of Bio-Resources in Yunnan at Yunnan Agricultural University.

## Conflict of Interest

The authors declare that the research was conducted in the absence of any commercial or financial relationships that could be construed as a potential conflict of interest.
